# Physicians Hand Book

**Published:** 1874-01

**Authors:** 


*A Physicians "Hand Book" for* 1874. By Drs. Wm. and A. D. Elmer. New York: W. A. Townsend.

The publisher of this Hand Book has been more ambitious than to produce a mere visiting list, but has comprised in his Hand Book a Visiting List, Book of Reference, Record of Practice and Account Book. In trying to combine so many things in one he has necessarily complicated them somewhat, and we think that beyond an ordinary visiting list it will find but little use.

